# Exploring the potential of Salmonella-mediated anti-sense RCAS1 RNA therapy in combatting aggressive breast cancer

**DOI:** 10.1016/j.omtn.2023.102089

**Published:** 2023-12-15

**Authors:** Amal Senevirathne, Irshad A. Hajam

**Affiliations:** 1 Chungnam National University, Yuseong-go, Daejeopn 34134, Republic of Korea; 2 Department of Pediatrics, University of California San Diego, San Diego, CA 92093, USA

## Main text

The study by Chandran et al.^1^ is an innovative and multifaceted approach involving targeted delivery, RNA interference, live-attenuated bacteria, and the complex interplay in breast cancer evolution and metastasis. Salmonella-mediated long non-coding (lnc) antisense RCAS1 (receptor-binding cancer antigen expressed on SiSo cells) RNA therapy showed a significant reduction in breast tumor volumes and completely arrested the metastatic lung lesions via mechanisms involving suppression of tumor-promoting genes, reduction in tumor-associated suppressor macrophages (TAMs), and efficient activation of T cell responses.^1^

Cancer research is a dynamic and evolving field, and innovative approaches to treat cancer are continually emerging. The multifactorial nature of cancer limits the effectiveness of a single treatment modality, and recent bacterial therapies in conjunction with radio- or chemotherapy have shown synergistic antitumor effects in preclinical animal models.^2^^,^^3^ The ability to precisely deliver therapeutic agents to cancer cells while sparing healthy tissues is crucial for reducing side effects and improving the treatment efficacy. This innovative Chandran et al.^1^ study tackles cancer specificity by engineering the *Salmonella* Typhimurium (ST) strain and delivering antisense RNA to silence RCAS1 mRNA that is highly expressed in cancer cells and tumor tissues.^4^ Both strategies could serve to increase target specificity while minimizing the effects on non-target tissues. In contrast to mice models, humans frequently encounter Salmonella bacteria and thus harbor bacteria-specific immunity.^5^ The major caveat to Chandran et al.’s^1^ study is the lack of investigational studies in prior Salmonella-exposed mice models. Investigating the effect of preexisting Salmonella-specific immunity on the efficacy of Salmonella-based therapies might offer significant insights into why such therapies have been met with little success in human clinical trials.

The use of bacterial systems to treat cancers goes way back, 100 years, to when bone surgeon Willian B. Coley successfully increased patient survival by *Streptococcus pyogenes* injection into inoperable bone and soft tissue cancer.^6^ Bacterial-mediated anticancer therapy has recently become a hot research topic, and several genetically attenuated bacterial species have been investigated to treat cancers.^3^ Among bacterial species, ST is a well-studied and effective anticancer bacterium investigated in experimental settings. ST preferentially replicates in poorly perfused necrotic and hypoxic tumor environments,^3^ which partly limits the efficacy of chemotherapeutic agents. Furthermore, being radioresistant, combinatorial therapies involving Salmonella bacteria with radio- or chemotherapy significantly inhibited tumor growth in preclinical animal models.^3^ lncRNAs represent a heterogeneous pool of transcripts that regulate multiple biological processes across a diverse range of tissues, and the use of lncRNAs as therapeutic agents is a cutting-edge strategy that is under rapid development.^7^ In this context, the idea of “antisense RNA against RCAS1” implies a highly targeted approach against a specific molecular target that is significantly associated with cancer. RCAS1 is known to play a significant role in immune modulation and strongly correlates with a poor prognosis in several types of cancer.^4^^,^^8^ Thus, the inclusion of live-attenuated ST as an antisense RNA carrier may offer a potential strategy to treat cancers effectively.

**Figure 1:**
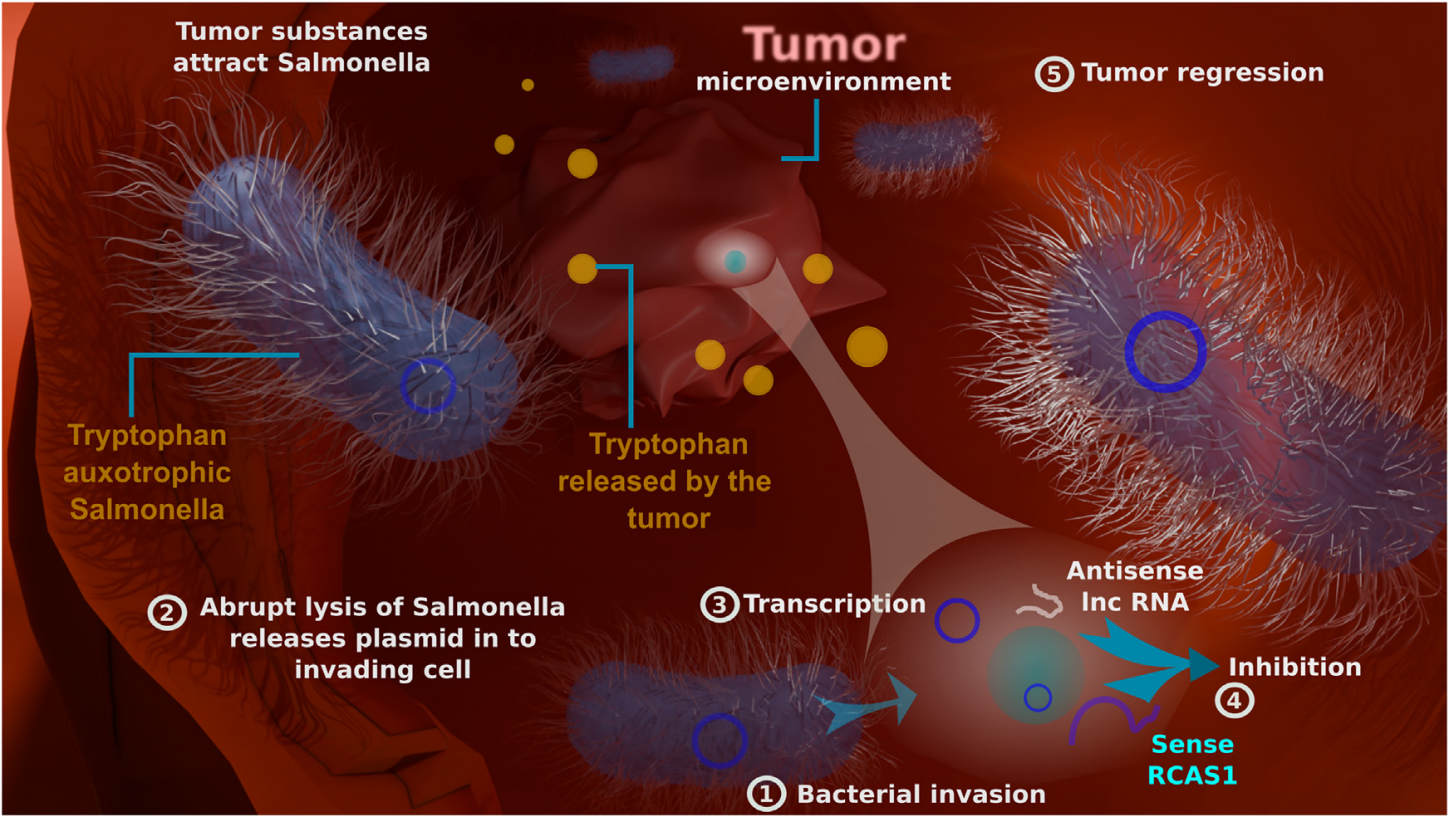
Major role of Salmonella-mediated antisense RCAS1 RNA therapy Tryptophan-auxotrophic *Salmonella* (ΔtrpA, ΔtrpE, Δasd+asRCAS1) utilizes tryptophan, synthesized and released by cancer cells into the TME, facilitating *Salmonella’s* survival. The therapeutic plasmid that encodes for lnc antisense RCAS1 RNA is released into cancer cells by abrupt lysis of *Salmonella*. The expression of antisense RNA reduces or aborts the activity of target RCAS1 mRNA expression, leading to regression of tumor growth. Native immunogenic components of *Salmonella* stimulate host immune responses favoring anticancer immunity.

The Chandran et al. study^1^ has successfully constructed a live-attenuated tryptophan-auxotrophic ST strain that selectively invades and propagates in cancer tissues (Figure 1). This can be attributed to Salmonella’s ability to grow under low-oxygenated environments by harnessing the resources from the tumor microenvironment (TME). It is possible to speculate that both Salmonella bacteria and tumor cells are in competition for resources such as tryptophan in the TME, thus making it an excellent choice to selectively deliver anticancer cargo to the TME.

The ultimate objective of any cancer treatment is to inhibit tumor growth and prevent metastasis. Thus, Chandran et al.^1^ tested an ST strain carrying the antisense RCAS1 plasmid in a murine model with 4T1 mouse-adapted breast tumor. The plasmid cargo carried within the Salmonella bacteria is intriguing. The use of lncRNAs as a therapeutic tool is a burgeoning area of research, and various strategies have been attempted for its therapeutic implementations.^7^ However, the use of tryptophan-auxotrophic ST for RNA delivery purposes is the first attempt of its kind.^1^ The tryptophan auxotrophy ensures the safety of this approach by limiting bacterial growth in healthy tissues, where tryptophan is less abundant compared to cancer tissues. Moreover, the choice of 4T1 breast tumors as a model is noteworthy. The 4T1 model is known for its aggressive behavior and ability to metastasize, making it a robust system for studying interventions that target both primary tumors and metastatic lesions.

The Chandran et al. study^1^ investigated the effect of Salmonella-mediated RCAS1 therapy on TAMs, which account for nearly 30%–50% stromal cells in the TME. TAMs exhibit an immunosuppressive M2-like phenotype and play a significant role in propagating tumor growth and metastasis.^9^ TAMs strongly express RCAS1 protein,^8^ and consequently, targeting TAMs would represent a powerful antitumor strategy to aggressively treat cancers. Notably, the Chandran et al. study^1^ reported a significant reduction in TAMs, and based on the gene expression studies, Salmonella-based antisense RCAS1 therapy significantly reduced immunosuppressive cytokines in the TME and downregulated genes related to cancer cell proliferation, migration, vascularization, and metastasis. RCAS1 is known to suppress cytotoxic T cells and natural killer (NK) cells via apoptosis of these mononuclear cells.^10^ Thus, Chandran et al.’s^1^ study measured T cell responses post-Salmonella therapy. The authors reported a significant increase in T cell responses, indicating that Salmonella-mediated RCAS1 targeting helps to overcome the tumor evasion mechanisms. Furthermore, Salmonella-mediated RCAS1 targeting completely arrested the metastatic lung lesions. If the findings reported by Chandran et al.’s^1^ study prove robust and reproducible, the implications for cancer therapy could be substantial.

Although Salmonella*-*based therapies have shown promising results in preclinical studies, translating this approach to the clinic requires addressing safety concerns, including potential side effects and immune responses, and future studies in prior Salmonella-exposed experimental animal models. Humans frequently encounter Salmonella bacteria and harbor bacteria-specific systemic antibody and T cell responses.^5^ We strongly speculate that preexisting Salmonella-specific immunity negatively impacts the antitumor efficacy of Salmonella-based therapies. Thus, future studies should be thoroughly conducted in bacteria-exposed animal models. Such studies will inform us on the rationale design of Salmonella-based therapies for human use.

In summary, a tumor-targeted delivery approach combining RNA interference and live-attenuated bacteria may offer a unique and powerful strategy for treating breast cancer malignancies. As with any groundbreaking research, the devil lies in the details, and further exploration of the mechanisms, safety, and efficacy of this approach in diverse preclinical models is warranted. If successful, this strategy could open new avenues for the development of advanced, targeted therapies for breast cancer and potentially other types of difficult-to-treat cancers. The ongoing research and subsequent publications stemming from this work will be eagerly awaited by the scientific community, clinicians, and patients alike.
